# Rapid Reversal of Chondroitin Sulfate Proteoglycan Associated Staining in Subcompartments of Mouse Neostriatum during the Emergence of Behaviour

**DOI:** 10.1371/journal.pone.0003020

**Published:** 2008-08-20

**Authors:** Hyunchul Lee, Catherine A. Leamey, Atomu Sawatari

**Affiliations:** Discipline of Physiology, School of Medical Sciences and the Bosch Institute, University of Sydney, Sydney, Australia; Medical College of Georgia, United States of America

## Abstract

**Background:**

The neostriatum, the mouse homologue of the primate caudate/putamen, is the input nucleus for the basal ganglia, receiving both cortical and dopaminergic input to each of its sub-compartments, the striosomes and matrix. The coordinated activation of corticostriatal pathways is considered vital for motor and cognitive abilities, yet the mechanisms which underlie the generation of these circuits are unknown. The early and specific targeting of striatal subcompartments by both corticostriatal and nigrostriatal terminals suggests activity-independent mechanisms, such as axon guidance cues, may play a role in this process. Candidates include the chondroitin sulfate proteoglycan (CSPG) family of glycoproteins which have roles not only in axon guidance, but also in the maturation and stability of neural circuits where they are expressed in lattice-like perineuronal nets (PNNs).

**Methodology/Principal Findings:**

The expression of CSPG-associated structures and PNNs with respect to neostriatal subcompartments has been examined qualitatively and quantitatively using double-labelling for *Wisteria floribunda* agglutinin (WFA), and the μ-opioid receptor (μOR), a marker for striosomes, at six postnatal ages in mice. We find that at the earliest ages (postnatal day (P)4 and P10), WFA-positive clusters overlap preferentially with the striosome compartment. By P14, these clusters disappear. In contrast, PNNs were first seen at P10 and continued to increase in density and spread throughout the caudate/putamen with maturation. Remarkably, the PNNs overlap almost exclusively with the neostriatal matrix.

**Conclusions/Significance:**

This is the first description of a reversal in the distribution of CSPG associated structures, as well as the emergence and maintenance of PNNs in specific subcompartments of the neostriatum. These results suggest diverse roles for CSPGs in the formation of functional corticostriatal and nigrostriatal connectivity within the striosome and matrix compartments of the developing caudate/putamen.

## Introduction

The ability to perceive and act on the external world emerges during distinct epochs in postnatal development. In rodents, the refinement of motor behaviour as well as the onset of coordinated sensory responsiveness has been reported to occur around 2 weeks after birth [1]–[3]. This period coincides with the consolidation of cortico-cortical [4], [5], as well as cortico-subcortical [6], [7] connections within the developing brain. Cortico-striatal and nigrostriatal connections are of particular interest as these networks are considered vital for the integration of sensory information [8], [9], goal directed learning [10], [11], self-initiated action [12]–[16], and the selection and execution of motor programs [17]–[20]. How these projections mature, and how they relate to the emergence of deliberate, coordinated motor behaviour is the subject of intense investigation.

The neostriatum, the rodent homologue of the primate caudate/putamen, is the main recipient of cortical as well as dopaminergic input within the basal ganglia. This input nucleus can be subdivided into histologically-defined compartments (the striosomes and the matrix) [21], [22], which in turn have been shown to receive input from disparate, albeit overlapping cortical areas [23]–[28] (see Discussion). The coordinated activation of these distinct corticostriatal pathways is thought to be vital for the execution and control of motor and cognitive behaviour. Tracing studies have indicated that corticostriatal projections are present at birth in the developing mouse brain. These axons appear to target neostriatal subcompartments soon after, with afferents from the cortex forming patches that overlap with tyrosine hydroxylase (TH)-positive clusters within the first week of postnatal development [7], [29]. The early precision of corticostriatal patterning, which precedes the onset of detectable functional connections [30], and a period of intense synaptogenesis within the caudate/putamen [31], suggests that these axons may rely on activity-independent mechanisms, such as molecular guidance cues, to reach their neostriatal targets.

Nigrostriatal terminals also exhibit changes in patterning across a similar period of postnatal development. Dopaminergic axons from the substantia nigra pars compacta (SNc) begin to innervate the caudate/putamen prenatally and start forming clusters a day before birth in mice [32]. These TH-positive nigrostriatal clusters initially overlap with μ-opioid receptor expressing patches, which label the striosome compartment within the striatum [33], [34]. This overlap is maintained until roughly the beginning of the second postnatal week, when the patchy distribution of nigrostriatal terminals yields to give a more diffuse innervation of the entire neostriatum by dopaminergic fibres [32].

The fact that the time-course of innervation and consolidation of both corticostriatal and nigrostriatal fibre pathways to specific subcompartments within the neostriatum occur before the onset of detectable functional connections within the caudate/putamen [30] indicates that this selective targeting may be regulated by shared activity-independent mechanisms. Chondroitin sulfate proteoglycans (CSPGs) are a member of a family of extracellular matrix (ECM)-associated glycoproteins that have been shown to regulate axon guidance [35]–[38], as well as play a role later in development in the form of perineuronal nets (PNNs): these extracellular matrix structures composed of CSPGs and other glycoproteins [39]–[42] are considered vital for the consolidation of neural circuits [43]. Previous studies have revealed that neurocan, a type of CSPG, along with related glycoproteins, are expressed transiently at the boundaries of neostriatal subcompartments in early postnatal development (within the first postnatal week) [44], [45], implicating proteoglycans in the formation and patterning of striatal circuits. PNNs, in turn, have been shown to begin forming throughout the developing mouse brain between the second and third postnatal week [46], [47]; recent evidence indicates that their expression is associated with the closing of the visual cortical “critical period” for monocular deprivation [43].

Although a distinct relationship between the neostriatal subcompartments and proteoglycan expression has been reported for the early postnatal rodent brain [44], [45], it has yet to be determined whether this or related patterns are maintained into adulthood. Moreover, the relationship between the expression of PNNs and the subcompartments of the developing neostriatum is not known. Revealing the dynamics of ECM proteoglycans in the caudate/putamen may provide insight into the role of these molecules in the formation and plasticity of functional corticostriatal and nigrostriatal circuits.

As a first step in this process, we examined the expression of CSPG-related structures in the neostriatum of young adult mice by staining for *Wisteria floribunda* agglutinin (WFA), a plant lectin reported to selectively bind to N-acetylgalactosamine residues [48], associated with chondroitin sulfates (CSs) within the ECM of both cortical and subcortical brain areas [49]–[52]. To our surprise, we observed PNNs loosely clustered throughout the nucleus. Treatment with chondroitinase ABC (ChABC) abolished the WFA signal, confirming that the lectin was binding to CS-related structures. In order to further determine the relationship of WFA staining ECM bodies to neostriatal subcompartments, and how they change during the course of postnatal development, we performed double-immunolabelling for WFA, and the μ-opioid receptor (μOR), an established marker for the patch or striosome subregion of the caudate/putamen [34], at six different postnatal time points in mice: postnatal days (P)4, P10, P14, P21, P28–31, and P40. We find that at the earliest ages (P4 and P10) WFA-positive clusters overlap preferentially with the striosome compartment. By P14, these glycoprotein clusters disappear. PNNs on the other hand, which are first seen at P10, continue to increase in density and spread throughout the caudate/putamen as the brain matures. Remarkably, unlike earlier WFA labelling, the PNNs overlap almost exclusively with the matrix compartment of the neostriatum by P14. These results implicate diverse roles for CSPGs in the generation of functional corticostriatal and nigrostriatal connectivity within the different subcompartments of the developing mouse neostriatum.

## Results

### Characterisation of WFA reactivity in mature mice

**Figure 1:**
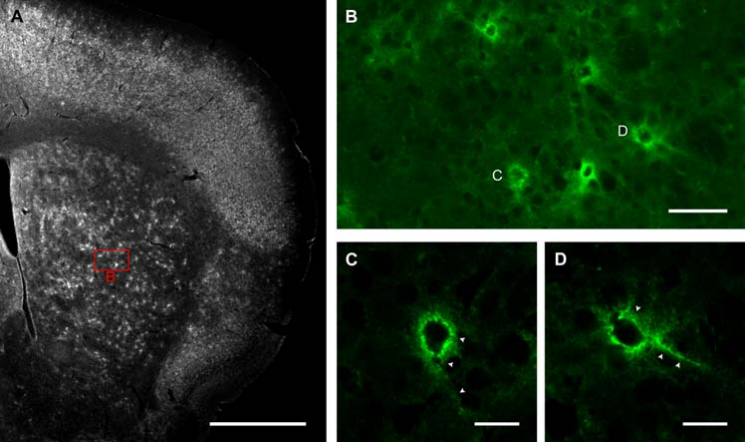
WFA labels PNNs. (A): Low-power image showing distribution of WFA labelling in a coronal section from a P31 mouse brain. Punctate labelling is visible in the striatum as well as in cortex and other brain areas. Scale bar: 1 mm. (B): Higher power confocal image of the region outlined with red in A, showing that the punctate labelling form PNN like structures. Scale bar: 50 μm. (C, D): Sample PNNs imaged at high power with confocal microscopy, correspond to those marked in B. The net-like labelling (arrowheads) surrounding the soma and proximal dendrites, which is characteristic of PNNs, is visible. Scale bar: 20 μm.

Three mice at 4–5 weeks of age (P28–31) were used to assess WFA expression in the neostriatum of near adult mice. To our surprise, the staining revealed a large number of punctate, WFA-positive structures loosely clustered throughout the entire striatum (Fig 1A, B). These structures exhibited the lattice-like characteristics typical of PNNs when imaged at higher magnification (Fig 1C, D) [42], [49], [53].

**Figure 2:**
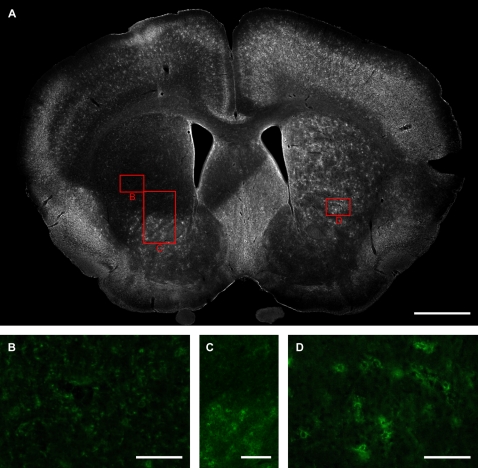
WFA labelling is abolished by ChABC. (A): Low power image of coronal section from a P31 mouse brain after injection of ChABC into the neostriatum of one hemisphere (left). Note the large region of decreased WFA labelling corresponding to the injection site. Robust labelling is present in the other hemisphere (right). Scale bar: 1 mm. (B–D): Higher-power images of labelled areas outlined in red boxes. The WFA staining is markedly reduced (B) compared to a corresponding region on the untreated side (D). The effect of ChABC on WFA staining is particularly apparent at the border of digested (upper) and undigested (lower) regions (C). Scale bar: 100 μm (B, D), 200 μm (C).

The abundance of PNN-like structures was surprising given that previous studies have indicated only sparse WFA-positive PNN staining in the corresponding nucleus of rats [54]–[56]. To ensure that the observed staining was specific, we performed injections of ChABC, an enzyme that cleaves CS-like glycosaminoglycan chains, into the neostriatum of one hemisphere. The ChABC completely abolished the WFA-positive PNN-like structures across much of the striatum in the injected side (Fig. 2A). Higher power analysis shows that the PNN-like structures were completely abolished by the treatment (Fig. 2B); clear boundaries between digested and undigested regions were also apparent (Fig. 2C). The untreated hemisphere showed robust staining as before (Fig. 2A, D). This demonstrates that the observed lectin binding is specific and CS-related. The appearance of the WFA-labelled structures and their sensitivity to ChABC indicates strongly that the observed structures are indeed PNNs.

Given these findings, it was of interest to determine how PNNs are related to the functional subcompartments of the striatum and the ontogeny of their appearance. To achieve this, three mice at each of six age points (P4, P10, P14, P21, P28–31, and P40) were used to qualitatively and quantitatively describe the relationship between WFA staining and striosome/matrix boundaries within the developing neostriatum. We focused our analysis on the anterior part of the striatum (from the rostral pole up to the crossing of the anterior commissure) due to the relatively high concentration of striosomes in this region. At each stage investigated, two representative sections taken from corresponding regions of the rostral portion of the caudate/putamen (rostral to the inter-hemispheric crossing of the anterior commissure and the globus pallidus (GPe)) are shown to illustrate the consistency of the relationship between proteoglycan expression and striatal subcompartments. For the quantitative analysis, these same two sections as well as an additional section, from a region located intermediate to the two displayed images, were used. Our material reveals dynamic changes in the relationship between CSPGs and the functional subcompartments of the neostriatum across early postnatal development.

### WFA-positive ‘patches’ overlap with striosomes in early postnatal development

**Figure 3:**
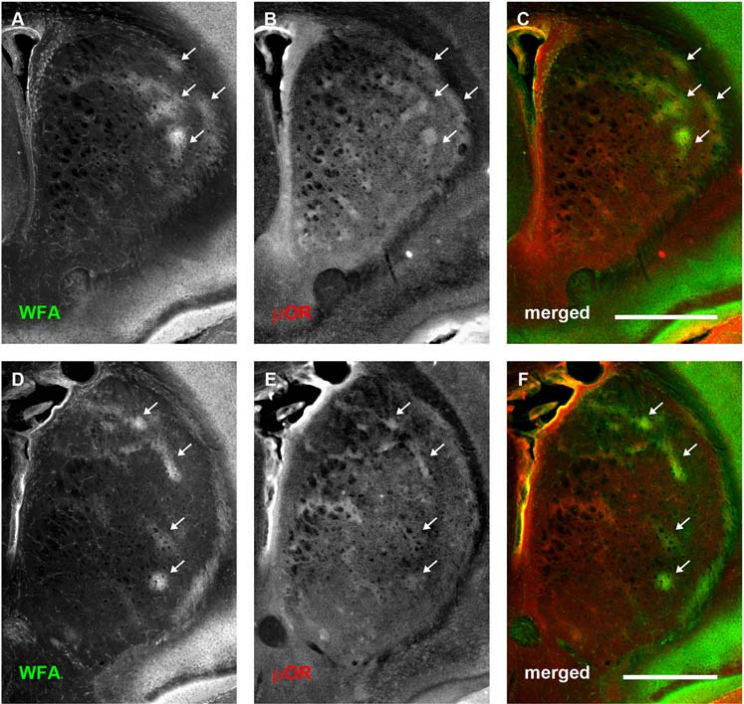
WFA-positive clusters overlap predominantly with μOR-positive striosomes at postnatal day 4. Each row shows, from left to right, the same neostriatal coronal micrographs revealing the distribution of WFA (A, D), μOR (B, E), and a merged image of the two (C, F). (A–C): Representative rostral section. (D–F): A more caudal section (level with anterior commissure). WFA staining is seen in large, amorphous ‘patches’ or clusters (A, D). Comparison with μOR staining (B, E) shows that WFA stained clusters overlap considerably with striosomes at this stage of development (arrows). This tendency is observed across the entire anterior striatum. The overlap can be clearly seen in the merged images where WFA staining is shown in green and μOR in red (C, F). In contrast to older mice, no PNNs are visible at this stage of development. Dorsal is up, lateral to the right. Scale bar: 600 μm.

**Figure 4:**
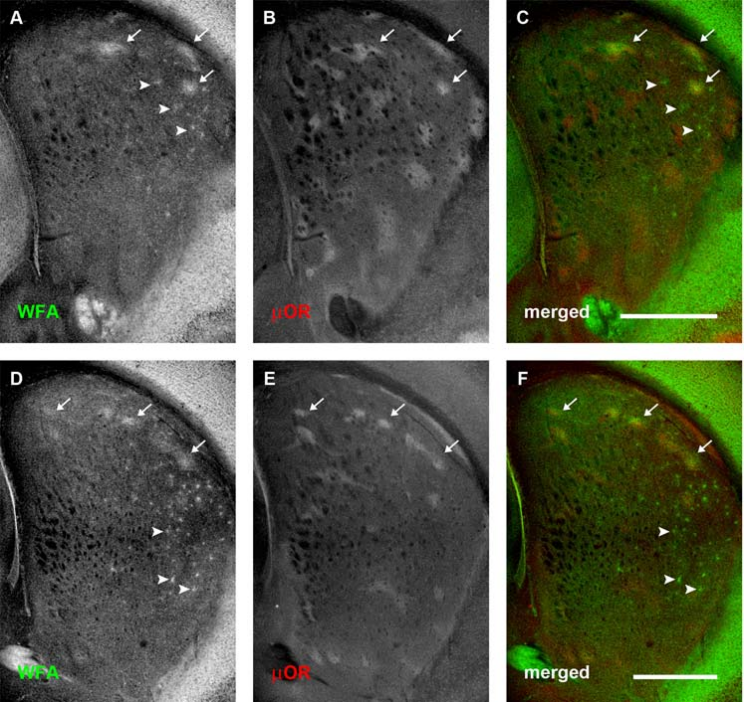
WFA staining changes in relation to μOR distribution at postnatal day 10. Conventions are the same as for Fig 3. (A–C): Rostral-most section. (D–F): More caudal section. (A, D): WFA distribution. (B, E): μOR distribution. (C, F): merged image. WFA ‘patches’ are mainly confined to the dorsal part of neostriatum, where they still overlap considerably with μOR defined striosomes (arrows). Postnatal day 10 also marks the appearance of punctate labelling which indicate PNN formation in the dorsolateral striatum rostrally (arrowheads; A). More caudally, PNNs are also seen in the lateral striatum and appear more numerous (D). PNNs are mainly confined to the matrix compartment of the neostriatum. Scale bar: 600 μm.

**Figure 5:**
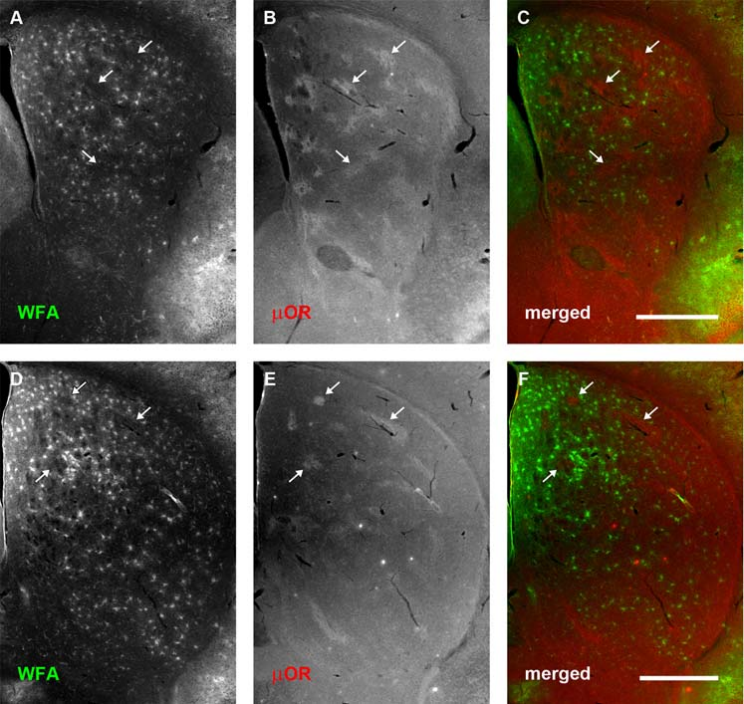
PNN density increases dramatically at postnatal day 14. Conventions are the same as for Fig 3. (A–C): Rostral sections. (D–F): More caudal sections. (A, D): WFA staining. (B, E): μOR distribution. (C, F): merged. At postnatal day 14, there is a dramatic increase in the number of PNNs: these are found to be distributed selectively within the matrix compartment. There is a marked paucity of proteoglycan expression in the striosome compartment (arrows). A high intensity of diffuse WFA staining associated with the extracellular matrix can be observed in the medial and dorsomedial portions of the neostriatum. Scale bar: 600 μm.

Relatively large amorphous ‘patches’ were observed in the earliest developmental time points examined. These structures were most apparent at P4, where clustered WFA labelling could be observed mainly within the dorsolateral region of rostral neostriatum (Fig 3A, D), although they continued to be expressed through P10 (Fig 4A, D). These WFA-positive patches were no longer detectable by P14 (Fig 5A, D).

**Figure 6:**
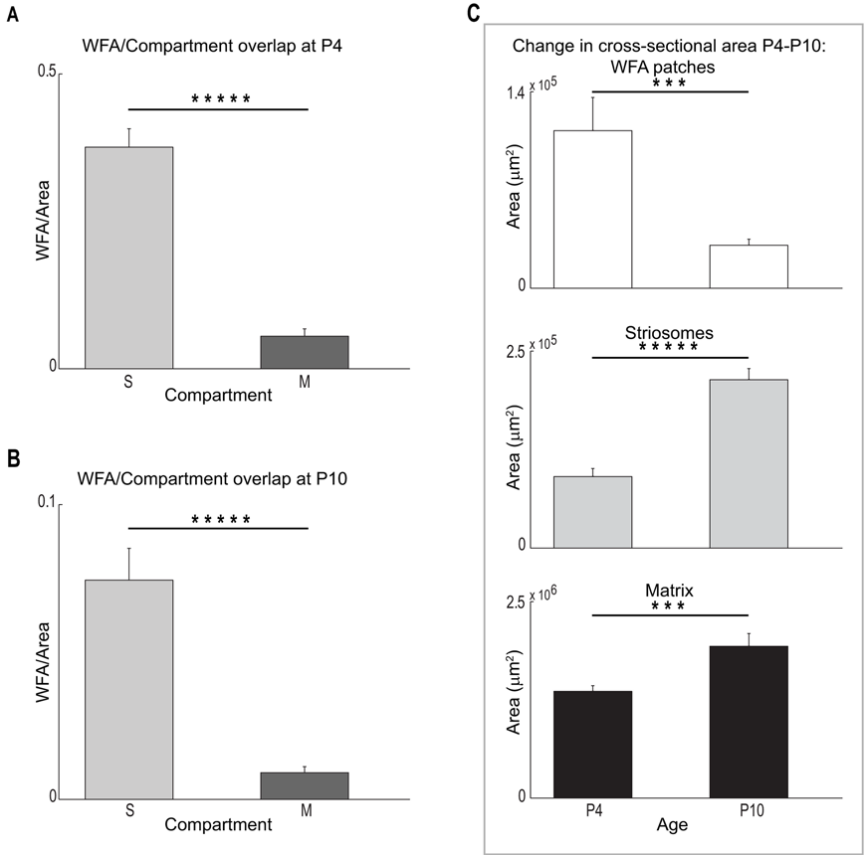
Quantitation demonstrates the overlap of WFA ‘patches’ and striosomes decreases during early postnatal development. (A) Mean area of overlap between WFA clusters and striosomes (S) or matrix (M) compartments at P4. Cross-sectional overlap is significantly greater in striosomes (Student's *t*-test; P = 5.2247×10^−8^), indicating that most of the clusters are situated in this neostriatal subcompartment at this age. (B) Overlap between WFA clusters and S and M compartments at P10. Although ‘patches’ remain overwhelmingly associated with striosomes (Student's *t*-test; P = 2.1063×10^−5^), the absolute area of overlap with both neostriatal subcompartments has dropped significantly by this time compared with P4 (note difference in scale for A and B; Two-way ANOVA, age and subregion as factors; F(1, 32) = 96.65, P = 3.4327×10^−11^ across ages; F(1, 32) = 119.2, P = 2.5329×10^−12^ across subregions; F(1, 32) = 52.2, P = 3.3113×10^−8^ for interactions between age and subregion). (C) Significant differences in overlap with age can be attributed to both an absolute decrease in the summed area of the WFA ‘patches’, as well as an increase in size of the neostriatum. (Top panel) Cross sectional area taken up by WFA clusters decreases significantly between P4 and P10 (*t*-test; P = 0.0037). Simultaneously, both striosomes (Middle panel) and matrices (Bottom panel) grow in size during this same period of development. The change in size in both cases is significant (S: *t*-test; P = 2.8080×10^−6^; M: *t*-test; P = 0.0053). Symbols (***) and (*****) indicate significance levels below P<0.01 and P<0.00001 respectively.

At P4, double staining with anti-μOR antibody (Fig 3B, E) revealed that many of these WFA ‘patches’ overlapped with striosomes (Fig 3C, F). Quantitative assessment of the overlap between these proteoglycan associated clusters and subregions within the neostriatum agreed with our qualitative observations. WFA patches overlapped with striosomes to a significantly greater degree than with the matrix (mean normalised overlapping areas with striosomes: 0.3758±0.0312; mean normalised overlapping areas with matrix: 0.0551±0.0125; Student's *t*-test: P = 5.2247×10^−8^; Fig 6A). In order to determine whether the CSPG-related patches represent specific binding of the lectin, we treated one hemisphere of P4 mice with ChABC. The CSPG-associated patches were digested following the treatment (data not shown) as demonstrated above for older animals.

The overlap between CSPG-related patches and striosomes continued to be observed at P10; as at P4, the densest WFA labelling overlapped with μOR-rich striosomes in dorsolateral regions of the neostriatum (Fig 4C, F). Quantitative comparisons of the overlap between WFA patches and neostriatal subregions indicated that the significant ‘preference’ of the glycoprotein clusters for striosomes was maintained through this developmental time point (mean normalised overlapping areas with striosomes: 0.0743±0.0108; mean normalised overlapping areas with matrix: 0.0091±0.0020; Student's *t*-test: P = 2.1063×10^−5^; Fig 6B).

When comparing across the two ages (P4 and P10), our analysis revealed that the degree to which CSPG-associated clusters overlapped with both striosome and matrix subregions decreased significantly (Two-way ANOVA, age and subregion as factors; F(1, 32) = 96.65, P = 3.4327×10^−11^ across ages; F(1, 32) = 119.2, P = 2.5329×10^−12^ across subregions; F(1, 32) = 52.2, P = 3.3113×10^−8^ for interactions between age and subregion). The presence of a strong interactive component indicates that the change in WFA overlap with neostriatal subregions is age-dependent. Measured values for cross-sectional areas in our sample suggest that this age-dependent change may be due to growth , and thus an increase in the size of the subregions within the neostriatum (Fig 6C, middle and bottom panels), as well as a decrease in WFA clustering (Fig 6C, top panel). The cross-sectional areas of the WFA ‘patches’ (decrease: *t*-test; P = 0.0037), striosomes (increase: *t*-test; P = 2.8080×10^−6^), and matrix (increase: *t*-test; P = 0.0053), all exhibit significant changes between these two age points.

### PNN expression begins at P10 and is associated with the matrix compartment

**Figure 7:**
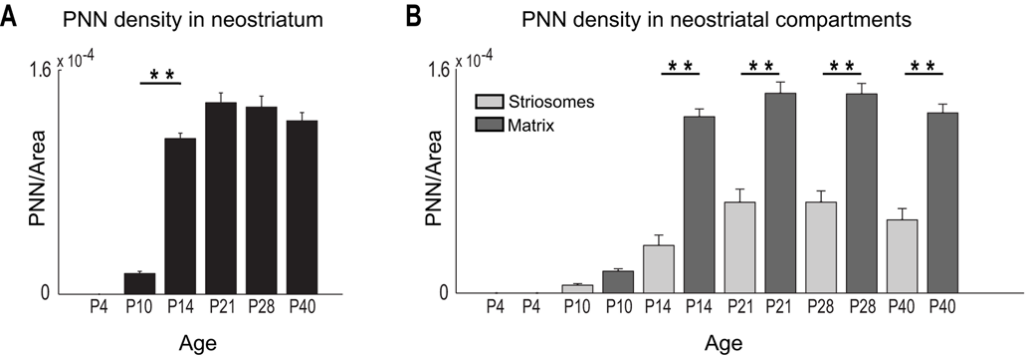
PNNs overlap selectively with the neostriatal matrix in postnatal mice. (A) PNN density increases significantly between P10 and P14 within the entire neostriatum; after P14, PNN density levels off and remains consistent into adulthood (one-way ANOVA, Tukey-Kramer test; F(5, 48) = 142.14, P<<0.00001). Bar indicates significant increase in PNN density between P10 and P14. (B) PNN density is significantly greater in matrix compared to striosomes for all ages after P14 (Two-way ANOVA, age and subregion as factors; F(5, 96) = 112.8, P<<0.00001 across ages; F(1, 96) = 236.62, P<<0.00001 across subregions; F(5, 96) = 20.15, P<<0.00001 for interactions between age and subregion). (**) indicates significance levels of P<<0.00001.

The youngest age at which we observed PNNs was at P10 (Fig 4A, D). Most PNNs at this age were restricted to the dorsolateral regions of the neostriatum. There also appeared to be more PNNs present in caudal (Fig 4D) compared to more rostral areas (Fig. 4A). This changed rapidly, however, with PNN expression extending throughout the caudate/putamen by P14 (Fig 5). The higher prevalence of PNNs in more caudal sections disappeared by P14, suggesting a caudorostral maturational gradient rather than a differential distribution across the striatum. The increase in PNN density (normalised to the cross-sectional area of the neostriatum) between P10 and P14 was highly significant (one-way ANOVA, Tukey-Kramer test; F(5, 48) = 142.14, P<<0.00001; Fig 7A). In addition to the increase in PNNs themselves, as the caudate/putamen matured, a diffuse WFA labelling of extracellular matrix emerged. At P14 this was most obvious dorsomedially (Fig 5C, F).

**Figure 8:**
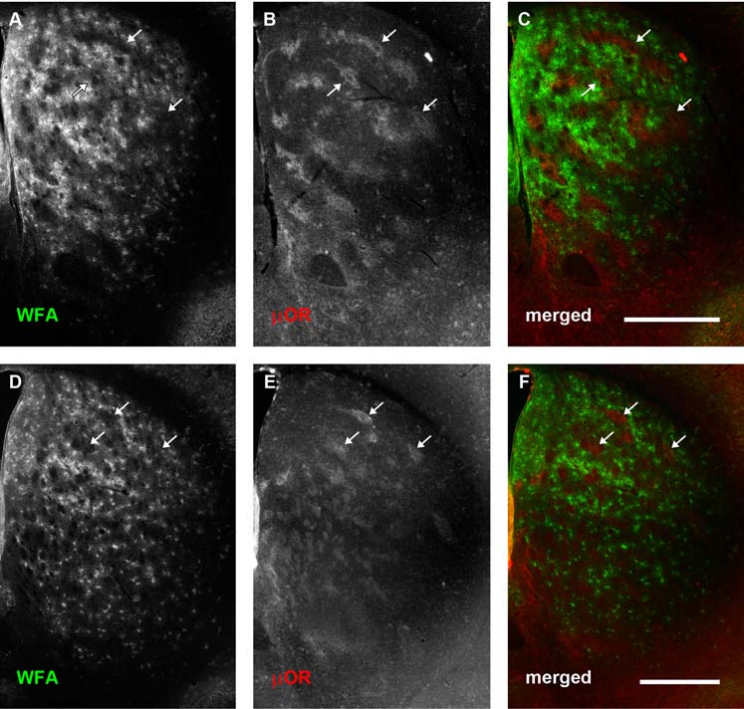
PNN distribution is maintained at postnatal day 21. Conventions are the same as for Fig 3. (A–C): Rostral sections. (D–F): More caudal sections. (A, D): WFA staining. (B, E): μOR distribution. (C, F): merged. The emergent, selective expression of PNN in the matrix compartment, first observed at P14, as well as the generally higher degree of WFA staining in medial and dorsomedial extracellular matrix, is maintained at P21. Scale bar: 600 μm.

**Figure 9:**
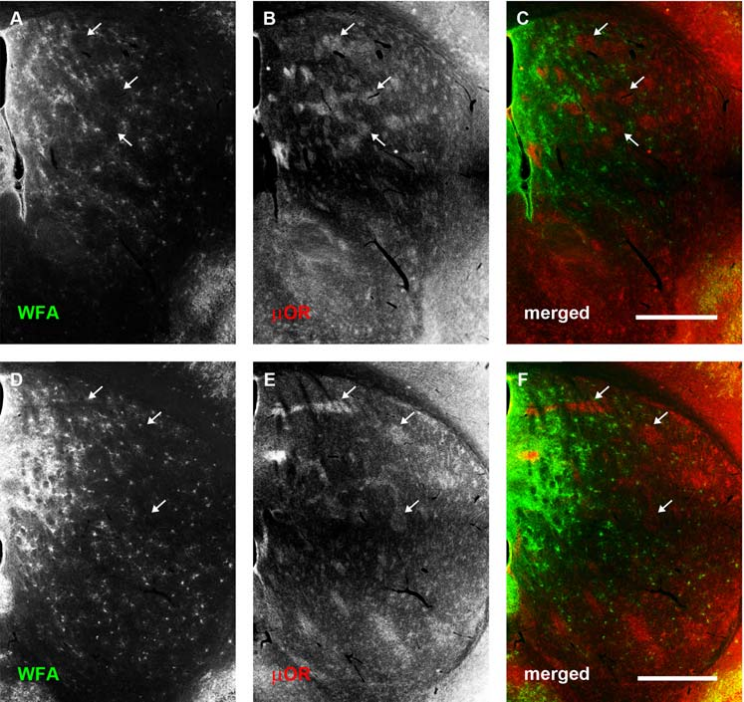
Pattern of WFA staining within the neostriatum continues into adulthood. Conventions are the same as for Fig 3. (A–C): Rostral sections. (D–F): More caudal sections. (A, D): WFA staining. (B, E): μ-opioid receptor distribution. (C, F): merged. Selective expression of PNNs in the matrix, as well as higher WFA staining in medial extracellular matrix continues into adulthood. Arrows indicate PNN-poor striosomes. Scale bar: 600 μm.

In contrast to the rapid increase in PNN formation between P10 and P14 (Fig 7A), the expression of PNNs changed little between P14 and P21 (Fig 8). The pattern was similar to that observed at P28–31 (Fig 1A and 2A) and continued into adulthood (P40, Fig 9). The PNN density also remained relatively unchanged into adulthood (Fig 7A). The diffuse extracellular WFA staining persisted and became more obvious in the dorsomedial striatum by P21 (Fig 8), but similar to the PNNs themselves, avoided the striosomes (striosomes appear as dark “islands” in a “sea” of WFA staining; see Fig 8 for example). This pattern also persisted into adulthood (Fig 9).

### PNNs form in the matrix compartment of the neostriatum

Double-labelling with the anti-μOR antibody and biotinylated WFA revealed that very few PNNs were present in striosomes (Fig 7B). In fact, a simple count of these proteoglycan structures indicated that over 90% of PNNs were found within the matrix compartment. This bias in expression was present from their earliest appearance at P10 (Fig. 4), and became even more obvious by P14 (Fig 5), when a rapid increase in PNN density was observed (see above).

Quantitation of the density of PNNs within striatal subregions confirmed the observed bias of the proteoglycan structures for the neostriatal matrix. At P10, a bias towards the matrix was seen but the difference was not significant (Fig 7B). At all later ages, PNN density (i.e., the number of PNNs normalized to the cross-sectional area of the respective neostriatal subcompartments) within the matrix subregion was significantly greater than within striosomes (Two-way ANOVA, age and subregion as factors; F(5, 96) = 112.8, P<<0.00001 across ages; F(1, 96) = 236.62, P<<0.00001 across subregions; F(5, 96) = 20.15, P<<0.00001 for interactions between age and subregion; Fig 7B). The strong interactive component indicated that the change in PNN densities observed between the two neostriatal subregions developed in an age-dependent manner. Remarkably, the formation of these perineuronal glycoprotein structures occurred in the matrix, the neostriatal subregion that exhibited little WFA staining earlier in development (see above).

### PNNs only partially overlap with TH expression in later stages of development

**Figure 10:**
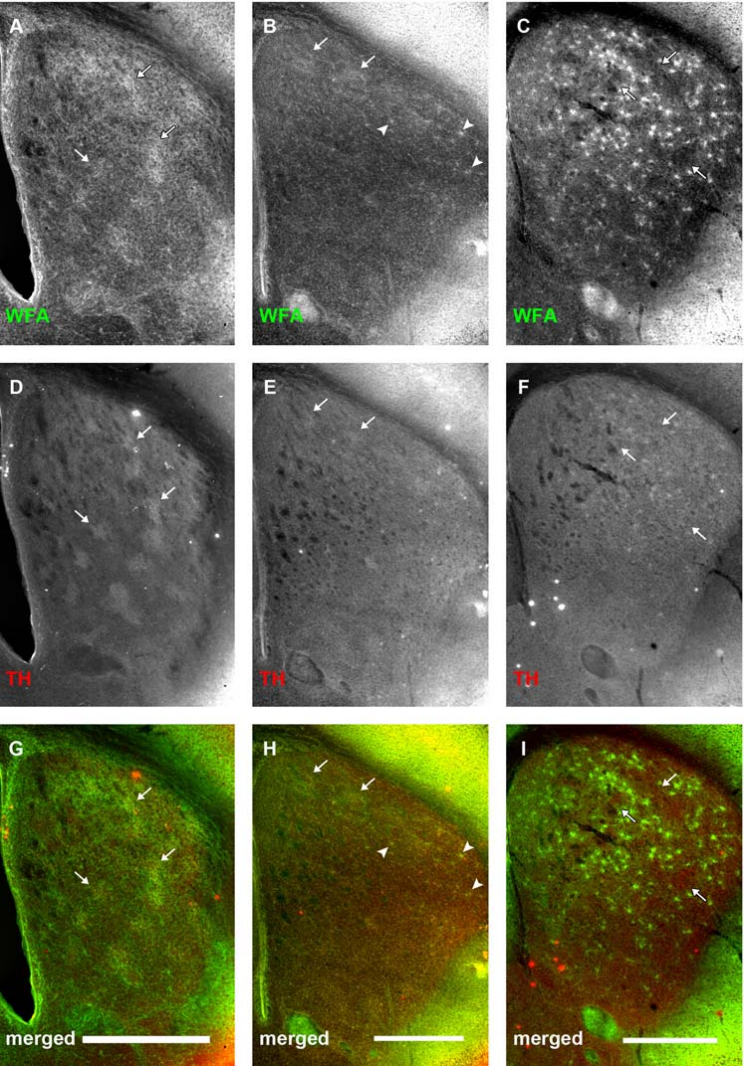
Early association between TH-positive striatonigral terminals and WFA staining dissipates after postnatal day 14. Panels show relationship between WFA binding (A–C) and tyrosine hydroxylase (TH) expression (D–F) at P4 (A, D, G), P10 (B, E, H) and P14 (C, F, I). (G, H, I) are merged channel images of the two corresponding images above. At P4, TH staining is seen in discrete patches. WFA clusters overlap well with these regions (A, D, G, arrows). At this age, TH-positive dopamine terminals correspond well with μOR-positive striosomes. By P10, TH staining has become considerably more uniform, especially in the medial regions of the neostriatum, than at P4, although some patches can still be observed dorsally. These remaining TH patches can be seen to overlap with WFA clusters (B, E, H, arrows). Arrowheads show PNNs starting to form in the dorsolateral striatum (B, H). By P14, TH staining is uniformly distributed in the entire neostriatum, when WFA staining is predominantly associated with PNNs in the neostriatal matrix (F). Arrows (F, I) mark WFA-poor areas which are reminiscent of striosomes. Scale bar: 600 μm.

Corresponding neostriatal sections from mice at different stages of early postnatal development were double-labelled with an anti-TH antibody and WFA to more directly observe the relationship between the developing nigrostriatal pathway and CSPG-associated WFA staining (Fig 10). Mice from three representative ages were used for this analysis: P4 (Fig 10A, D, G), when only WFA clusters that preferentially overlapped with striosomes were present; P10 (Fig 10B, E, H), the transitional age when WFA patches gave way to PNN formation in the matrix; and P14 (Fig 10C, F, I), the time point at which PNNs had already distributed across the striatal matrix.

At P4, WFA-positive patches overlapped considerably with TH clusters. This was consistent with previous observations [44], [45], including our own, of a correspondence between the clustering of CSPG-associated labelling (Fig 10A) and the striosome compartment (as defined by both μOR expression (see above), and nigrostriatal terminals; Fig 10D, G; [44], [45]). By P10, the TH staining had become more uniformly distributed, especially in the dorsolateral regions of the neostriatum, although some TH-positive patches could still be observed (Fig 10E). Here too, there remained a correspondence between WFA expression and TH labelling: discrete TH clustering gave way as WFA patches became scarce. By P14, TH-positive patches were no longer present, and TH-positive fibres were uniformly distributed across the neostriatum (Fig 10F); TH expression remained high within the whole of the caudate/putamen into adulthood (not shown; [32], [34]). Similarly, PNNs formed across the entire neostriatum (Fig 10C). Unlike the TH labelling, however, the PNNs were not uniformly distributed (Fig 10I): they were largely absent from regions, which, as we have demonstrated (Fig 5, 8, and 9), correspond to μOR-defined striosomes. These data provide evidence for a specific interaction between the formation of proteoglycan related structures and the postnatal development of distinct subregions within the mouse neostriatum.

## Discussion

The current study demonstrates that the distribution of CSPG-related staining undergoes dramatic and rapid changes in the caudate/putamen during postnatal development. At the earliest time points examined, P4 and P10, WFA stains diffuse patches that preferentially overlap with μOR-expressing striosomes, especially in the dorsolateral neostriatum. This patchy labelling disappears by P14, however, only to be replaced by the formation of PNNs (first seen at P10), beginning at the dorsolateral region of the caudate/putamen. As the animal matures, these proteoglycan associated structures spread across the neostriatum. Remarkably, unlike the earlier CSPG-related patches which preferentially overlap with striosomes, the PNNs are almost exclusively expressed in the matrix compartment. This explicit reversal in WFA staining may underlie a transition in the role of these proteoglycans and related glycoproteins in the formation of functional connections between cortical and subcortical projections, and specific subsets of neurons within the neostriatum. To the best of our knowledge, this is the first description of a reversal in the distribution of CSPG-related staining from striosome to matrix subcompartments. It is also the first description of the emergence of PNNs in the neostriatum. The results have important implications for our understanding of the development and plasticity of striatal circuitry.

### Technical considerations

A combination of antibody staining and lectin binding were used to assess the localisation of neostriatal compartments and CSPG-associated structures respectively. Double-labelling using fluorescent stains is a well-established method for visualising different anatomical features in brain tissue (e.g., [46]). Although antibodies for μOR have not been used previously in combination with WFA to analyse the relationship between the expression of CSPG-related proteoglycans and neostriatal subcompartments (i.e., striosomes), we are confident that our observations are not the result of unexpected interactions between these specific reagents for two reasons: (1) in all our double-labelling experiments, the WFA was applied first, thus eliminating the possibility that binding of the μOR antibody may somehow block the reactivity of WFA with the CSPG associated structures; and (2) in cases where WFA was used alone (not shown), or in combination with antibodies to TH (Fig 10), a similar pattern of lectin binding was observed in the neostriatum. Indeed, at the earliest ages examined (P4 and P10), a clear overlap between μOR and WFA expression was observed with little indication of interference. Moreover, combined TH/WFA labelling at the same early time points was consistent with the CSPG patterning described in previous reports [44], [45] (although see below).

Second, the overlap between matrix and striosomes and CSPG-related labelling was determined by manually tracing or marking these structures. It is entirely possible that the subjectivity inherent in this method may provide an inaccurate estimate of the overlap between WFA staining and neostriatal subcompartments. Although artifactual deposits of primary μOR and or secondary fluorescent antibodies were encountered in a number of sections, they were easily distinguishable from the even, low-contrast labelling characteristic of striosomes (e.g. arrows in Fig 5E). Nevertheless, we cannot rule out the possibility that due to (1) incomplete labelling by either the antibody or the lectin, and (2) variable accuracy in the assessment of striosome/matrix borders, we have underestimated the extent of striosomes in our analysis. Although this may affect the actual overlap values we calculated from our samples, the general consistency of our observations across sections, animals, and ages argues that our results are both repeatable and reliable.

Our study has shown, for the first time, that robust CSPG-related staining decorates PNNs in the neostriatum of rodents. While this may be related to species differences and/or the type of CSPG examined (see below), it is possible that the immunohistochemical technique used here, tyramide signal amplification, which is particularly sensitive, has allowed us to demonstrate staining which was not apparent to other investigators. In fact, in our own hands, protocols which used different methods revealed much less robust staining of PNNs in the mature striatum which could have been readily over-looked. The fact that the observed WFA staining was sensitive to ChABC treatment makes us confident that the lectin binding is specific to CS-related structures. Recent work has suggested that while WFA reactivity typically co-localises with CSPGs [57] ), the nature of the binding may not necessarily be direct [40], [41]. For this reason, we refer to the staining observed as CSPG-associated/related, rather than as CSPGs *per se.*

### Early postnatal development: WFA patches overlap with striosomes

The early postnatal expression of CSPG-related structures in the mouse neostriatum described in this study bears some similarities with proteoglycan patterning at corresponding developmental time points described for the caudate/putamen of the rat [44], [45], with some notable differences. Both in mice and rats, proteoglycans exhibit a strong association with striosomes at least through P10/P11. It should be noted, however, that in rats, the CSPG structures appear to surround the subcompartment [44], [45], instead of exhibiting a clear overlap between striosomes and WFA labelling as we observed in the mouse. Moreover, after this time point, divergence is more apparent, with a variety of proteoglycans reported to eventually exhibit a diffuse pattern of labelling throughout the entire neostriatum in rats [44], [45]. In contrast, in mice, we observed a total disappearance of the early striosome-associated WFA-positive clusters by P14. This discrepancy may be due in part to the different types of proteoglycans and glycoproteins targeted by lectin binding as opposed to antibody staining. Different proteoglycans have distinct glycosaminoglycan chains, and previous work has shown that these glycoproteins can influence neurons in a type-dependent manner [36], [37], [58]. In the current study, WFA was used to identify CSPG-related structures in the developing neostriatum [49]. In the rat, monoclonal antibodies were employed in studies that focused on the expression patterns of specific proteoglycans in caudate/putamen [44], [45]. It is entirely possible that other proteoglycans with different glycosaminoglycan chains (e.g. keratin sulfate proteoglycans (KSPGs)) may also exhibit a different pattern of expression in the developing mouse neostriatum. Indeed, recent evidence suggests that WFA labelling only partially overlaps with brevican, a type of CSPG [40], indicating that even different CSPGs may exhibit varied CNS distributions. Previous work that employed WFA to label PNNs in developing rat brain, however, also revealed very little CSPG-related staining in P7 and P14 neostriatum [55], [59]. It is unclear whether the differences between these studies and our work are related to technical issues (mentioned above) or species differences.

Differences in cytoarchitecture and immunoreactivity have been reported for a variety of cortical and subcortical brain regions between rats and mice [34], [60], [61]. For example, although TH-positive nigrostriatal clusters remain visible into adulthood in rat dorsolateral neostriatum [32], [34], dopaminergic patches are no longer detectable by P14 in mice. Further studies will be required to determine whether the differences observed in early CSPG-related staining in the developing neostriatum of mice and rats are attributable to proteoglycan type, histological protocols and/or species dependent variability in the caudate/putamen.

### Postnatal development after P10: PNN expression in the matrix

Our results indicate a clear developmental progression of PNN formation in the mouse neostriatum. They were first observed in the dorsolateral region at P10, and spread medially and ventrally to cover the entire caudate/putamen by P14. This time of onset is consistent with previously studies showing that most cortical and subcortical structures begin expressing PNNs sometime between P7 and P14 [46]. To our surprise, the distribution of these CSPG-associated structures was restricted overwhelmingly to the matrix compartment. To our knowledge, this is the first description of PNN formation with respect to striosome/matrix boundaries. Previous work has demonstrated an explicit relationship between neostriatal striosomes and CSPG expression [44], [45] in early postnatal development (see above); earlier descriptions of proteoglycan distribution after the recession of this initial association, however, have not described the emergence of PNNs explicitly. As mentioned above, our ability to detect PNNs in mature and maturing striatum may be related to the sensitivity of the histochemical technique employed. The regional specificity (matrix over striosomes) and distributed patterning suggests that PNNs may play a role in the maturation of connections to specific cells that underlie sub-circuits within the developing neostriatum.

PNNs have been shown to be associated with both interneurons, particularly those that stain for the Ca^2+^-binding protein parvalbumin, and projection pyramids in various brain regions [49], [50], [62]–[64]. In the neostriatum, fast spiking interneurons have been demonstrated to express this Ca^2+^ binding protein [65]–[67] and PNNs have been reported to surround parvalbumin-positive cells in the caudate/putamen of primates [68]. Although parvalbumin-positive cells are rare (constituting roughly <1% of all neurons in the neostriatum [67]), they have been shown to provide the main source of cortically regulated “feedforward” inhibition within the striatum [69]–[73]. In our material, the percentage of WFA-positive cells is close to the expected values for fast-spiking interneurons within the caudate/putamen (1∼2%, calculated from the ratio of identified PNNs to estimated cell density within caudate/putamen sections [67]). Moreover, the developmental time-course and spatial spread of PNN-associated neurons is remarkably similar to what has been described for parvalbumin-positive cells in the caudate/putamen of the developing rat [74]. Previous work has described a strong high lateral to low medial gradient in the localisation of parvalbumin-positive cells [75]. The distribution of our PNN-labelled neurons only partially overlaps with this patterning. Other studies have indicated, however, that parvalbumin staining may under-represent the presence of fast spiking interneurons in the neostriatum [76]. In light of the facts that (1) PNNs have been implicated in buffering the extracellular environment of fast spiking neurons [53], and (2) our estimated percentage of PNN–associated cells is slightly greater than the expected density of parvalbumin expressing interneurons [67], PNNs may be associated with fast spiking striatal neurons *in toto*. Although further work will be required to reveal the identity of PNN-associated cells in the mouse caudate/putamen, the prevailing evidence indicates that at least some of the cells associated with these CSPG-related structures in the neostriatum are likely to be fast spiking interneurons.

### Postnatal development after P10: WFA expression in the extracellular matrix exhibits a high dorsomedial to low ventrolateral gradient

From P14 onward, a relatively high level of diffuse staining of the extracellular matrix was observed in a high dorsomedial to low ventrolateral gradient within the neostriatum. This patterning resembles the general nigrostriatal terminal distribution described in adult rat [32]. Although a similar patterning of dopaminergic axons has not been described in adult mice, it is possible that CSPG-related labelling may be revealing a thus far under-explored organisation of nigrostriatal projections in these rodents. Further experiments will be required to determine if this is the case.

### The changing role of CSPG-related structures in neostriatal development: the switch from striosomal WFA clusters to matrix PNNs

We observed a dramatic reversal in the formation of CSPG-related structures across neostriatal compartments during early postnatal development of the mouse neostriatum. This spatial/temporal transition argues for compartment specific, age-dependent roles for proteoglycans and related glycoproteins in the maturation of the caudate/putamen.

Axon guidance cues have long been known to play important roles in regulating connectivity in various parts of the nervous system, including the neostriatum [77]–[84]. Proteoglycans have also been demonstrated to influence axon guidance of cortical and subcortical afferents in cultured cells as well as *in vivo* [35]–[38]. The relationship between the initial appearance of CSPG-associated patches and the confinement of nigrostriatal and corticostriatal terminals to striosomes is not currently known. Comparison of our data with previous studies [7], [32], [85] shows that the overlap of CSPG-related patches with neostriatal striosomes persists after the initial patterned innervation of the caudate/putamen by both nigrostriatal and corticostriatal axons. While causality remains to be determined, it is possible that the proteoglycan-associated clusters provide a guidance cue for the latter to reach their striosomal target. Indeed, both nigrostriatal and corticostriatal axons continue to selectively target striosomes until soon after P10, which correlates well with the time at which the CSPG-related clusters disappear [7], [32].

Alternatively (or as well), the proteoglycan-associated clusters may help define a permissive environment for nigrostriatal and corticostriatal terminals to consolidate their connections with striosomal neostriatal neurons. CSPG-associated structures, such as PNNs, have been theorised to be important in buffering activity-dependent ionic fluctuations in the local environments of fast spiking cells [53]. It is possible that the large CSPG-related clusters overlapping with neostriatal striosomes act in a similar capacity by providing a stable environment, and ultimately facilitating activity-dependent modification and consolidation of connectivity between cortical afferents, dopaminergic projections, and striosomal cells in the caudate/putamen.

PNNs, unlike the CSPG-related clusters, are associated with individual neurons, rather than populations of neurons [49], [53], [63]. Nevertheless, these proteoglycan structures may very well be involved in similar pathfinding and/or regulatory roles, albeit at the single-cell level. Both corticostriatal and nigrostriatal axons continue to project into the neostriatum after the second postnatal week [7], [32], and it may be that the PNNs are guiding and consolidating these or other inter- and/or intra-striatal terminals to form functional connections with individual proteoglycan-associated cells [73], [86]. Corticostriatal projections are of particular interest, as previous work has demonstrated that parvalbumin-positive fast-spiking neostriatal interneurons, a possible candidate for PNN association, receive direct cortical input [75]. Synaptic activation as a result of cortical stimulation can also first be detected in these interneurons near the end of the second postnatal week [30], the same time period in which we see a precipitous increase in PNN expression. Nigrostriatal terminals may also be influenced by PNNs, as (1) CSPGs have been shown to have a chemoattractive influence on dopaminergic axons [87], and (2) both the appearance of PNNs and the growth of nigrostriatal afferents within the matrix occur at around the same time. In light of this, it is interesting to note that fast-spiking interneurons are known to respond to direct dopaminergic activation via D1 receptors [88], [89]. Curiously, D1 receptors also exhibit preferential expression in striosomes, especially early in postnatal development, when μOR expression coincides with the patchy distribution of TH terminals in the rat neostriatum [90].

PNN expression has been shown to demarcate the termination of the visual cortical ‘critical period’ for monocular deprivation during development [43]. The timing of PNN proliferation within the matrix introduces the possibility that these proteoglycan structures may be involved in a similar maturation process for the neostriatum as has been implicated in the cortex and other brain areas [46], [47], [55], [59], [91]–[93]. In the case of the caudate/putamen, the ‘closing’ of a neostriatal sensorimotor ‘critical period’ may depend on the consolidation of corticostriatal connections to fast-spiking neurons within the matrix.

In light of this possibility, it is of considerable interest that very few PNNs were observed within striosomes. Neostriatal subcompartments receive input from different (but overlapping) neuronal populations within cortical areas, with the matrix primarily receiving input from cells in superficial layers of sensorimotor cortex and callosally-projecting neurons in prefrontal areas, while the striosomes are targeted mainly by deep layer corticofugal neurons situated in prelimbic, infralimbic, and premotor cortices [23]–[28]. These subregions are also considered to influence motor and cognitive behaviour in different ways: the matrix gives rise primarily to the striatonigral and striatopallidal pathways which serve complimentary roles in the execution of behaviour, while output neurons originating from the striosomes, some of which preferentially target the substantia nigra pars compacta (SNc), assist in the selection and regulation of appropriate motor and cognitive commands [27], [94]–[104]. In this context, the presence of a regulatory neostriatal subcompartment that remains relatively “plastic” throughout the animal's lifetime (i.e., having less consolidated connectivity as indicated by proteoglycan expression) could provide an advantage in adapting to ever changing external and internal conditions. In support of this possibility, recent work has emphasised the critical role of dopaminergic neurons within the SNc, a recipient of input from a subset of medium spiny neurons (MSNs) within striosomes [95], [96], [105], in reinforcement learning and reward prediction [106]–[112]. The paucity of PNN formation in the striosomes may thus reflect the function of this neostriatal subcompartment with respect to behavioural control.

### CSPG-associated staining targets regulatory circuits throughout early postnatal development

Although the identity of PNN-associated neurons in the mouse striatum has yet to be determined, if we consider fast-spiking interneurons as a potential candidate, it is of interest to note that these cells and neurons associated with striosomes, the neostriatal compartment most associated with CSPG-related staining early on, share at least two things in common. First, they both act to regulate the inhibitory output of the feedback cortico-basal ganglia pathways, either by contributing to the disinhibition of GABAergic projection neurons in the substantia nigra pars reticulata (via activation of inhibitory neurons in the GPe) [101], [103], [104], and the possible activation of dopaminergic nigrostriatal neurons within the SNc [96], [105], as in the case of striosomal medium spiny neurons (MSNs), or by feedforward inhibition of neostriatal MSNs via direct cortical activation [70], [72], [73], as in the case of fast-spiking interneurons. Second, they both receive robust and specific input from corticostriatal as well as nigrostriatal axons [7], [32], [34], [73], [85], [95], [97]. Indeed, recent evidence indicates that aberrant neostriatal activity observed as a result of disrupting the nigrostriatal pathway is dependent on the influence of dopamine on both the striatonigral and striatopallidal pathways, as well as the fast spiking interneurons [113].

### Timing of afferent innervation implicates a potential instructive role in the formation of CSPG-related structures

Finally, the timing of corticostriatal and nigrostriatal innervation also implicates a possible causal role for these terminals in the formation of CSPG-associated structures. It would be of particular interest to determine the relationship between the appearance of CSPG-related clusters and the specific innervation of striosomes by corticostriatal and nigrostriatal terminals in the late embryonic/early postnatal period. Recent studies in cultured cells indicate that activity is necessary for the formation of PNNs [114], although the genesis of these structures appears to be independent of glutamatergic activity [115]. Given that nigrostriatal axons are present within the neostriatum before the arrival of corticostriatal terminals, it is possible that activity-dependent dopamine release in the caudate/putamen may provide an instructive signal in the formation of these potentially chemoattractive proteoglycan structures [37].

Although further studies will be required to determine the specific role CSPGs play in early postnatal development, the association of CSPG-related structures with essential regulatory components of the neostriatum suggests that they play a vital part in the proper wiring of key circuits within the motor/behavioural control system.

## Materials and Methods

### Animals and preparation of tissue

All procedures were performed on C57/BL6 mice, were approved by the Animal Ethics Committee of the University of Sydney and conformed to NHMRC guidelines. Mice from six age groups (3 each of P4, P10, P14, P21, P28–31, and P40 for a total of 18 animals) were deeply anaesthetised with Euthal and perfused transcardially with 0.9% saline followed by 4% paraformaldehyde+0.3% Bouin's fixative in 0.1 M phosphate-buffered saline (PBS). Brains were removed and postfixed overnight before being placed in 30% sucrose in 0.1 M PBS. Brains were then blocked and embedded in gelatin albumin hardened with glutaraldehyde. Coronal sections 60 μm thick spanning the rostrocaudal extent of the neostriatal head and body (corresponding roughly to1.7mm anterior to, and −1.1mm posterior to bregma (Paxinos and Watson, 2001)) were cut using a freezing microtome (Leica). Sections were collected in 0.1 M PBS. Alternate sections were Nissl stained.

#### Chondroitinase ABC injections

Three four to five week old mice (P28–P31) were anaesthetised with 2–4% isofluorane in oxygen and placed in a rodent stereotax (Kopf Instruments, USA). Injections of chondroitinase ABC (ChABC; protease free, 15 U/ml in 0.9% saline, Sigma) were made at 1.5mm lateral to bregma, at a depth of ∼2mm, with glass pipettes connected to a pressure injector (WPI, USA). Mice were euthanised and transcardially perfused 3 days post-injection (see above).

#### Immunohistochemistry

To characterise the relationship between WFA staining and the developing subregions within the neostriatum, the brain sections of animals from 6 representative age points (P4, P10, P14, P21, P28–31 and P40; 3 animals at each age) were processed for both a biotinylated *Wisteria floribunda* agglutinin (WFA, a lectin thought to bind to N-acetylgalactosamine in CS chains, but see also [40]) and an antibody to the μ-opioid receptor-1 (μOR, a marker for striosomes within the caudate and putamen).

Sections were washed in 0.1 M PBS prior to immunohistochemical procedures, then quenched in a mixture of 45% ethanol+0.3% hydrogen peroxide in 0.1 M PBS for 30 minutes. After washing in 0.1 M PBS, sections were incubated for 2 hours at room temperature or overnight at 4°C in biotinylated *Wisteria floribunda* lectin (WFA, Vector Labs) (10 μg/ml). Bound WFA was visualised using the fluorescein-conjugated TSA kit (Perkin Elmer) then washed several times in 0.3% Triton-X in 0.1 M PBS. To visualise striosome/matrix compartments, sections were then incubated in rabbit polyclonal anti μ-opioid receptor-1 antibody (Abcam; 1∶2000 in 0.3% Triton-X, 0.1 M PBS, 4% normal goat serum) for 1 to 2 nights at room temperature. After being further washed in 0.3% Triton-X in 0.1 M PBS, the primary antibody was revealed using goat anti-rabbit antibody conjugated to Texas red (Molecular Probes; 1∶200 in 0.3% Triton-X, 0.1 M PBS, 4% normal goat serum) for 2 hours at room temperature. Sections were washed then mounted on gelatin coated slides, coverslipped using Slow Fade anti-fade mountant (Invitrogen).

To characterise the relationship between CSPG-related staining and the developing nigrostriatal pathway, the brain sections of an additional three animals from three representative age points (P4, P10, and P14) were processed using WFA and a polyclonal antibody directed against tyrosine hydroxylase (TH).

Tissue preparation for WFA ligand binding was identical to the procedure described above. Sections were then incubated with a sheep polyclonal anti-tyrosine hydroxylase antibody (Abcam; 1∶500 in 0.3% Triton-X, 0.1 M PBS, 4% normal rabbit serum) overnight at room temperature. The primary antibody was revealed with donkey anti-sheep secondary antibody conjugated to Texas Red (Invitrogen, 1∶200 in 0.3% Triton-X, 0.1 M PBS, 4% normal rabbit serum). Sections were mounted and prepared for fluorescence imaging as described above.

#### Analysis

Sections were imaged using a Zeiss deconvolution microscope with an AxioCam HR camera or a Zeiss LSM 510 META confocal microscope for high resolution imaging. Analysis was confined to the rostral half of the striatum (anterior to globus pallidus, and the crossing of the anterior commissure). Three representative sections from corresponding regions of the striatum stained for WFA and μOR were used for quantitative analyses. Images were first cropped to isolate the neostriatum using adjacent Nissl sections as a reference. Cross-sectional area of the neostriatum, WFA ‘patch’ count and area, striosome count and area, and PNN count were determined using a commercially available cell and slice reconstruction software package (Neurolucida System; MBF). Two features, the WFA ‘patch’ count and area, and PNN count were used to compare the development of proteoglycan-associated structures within neostriatal compartments across the examined ages. To determine the degree of overlap between WFA ‘patches’ and striosome/matrix subregions, total WFA ‘patch’ cross-sectional areas were normalised to the total areas taken up by either the striosome (normalised WFA patch overlap-striosomes) or matrix subregions (normalised WFA patch overlap-matrix) within individual sections, depending in which region the lectin binding domains were observed to be co-expressed. Similarly, PNN counts were normalised to the surface areas occupied by the overlapping neostriatal subregions (normalised PNN overlap-striosomes and normalised PNN overlap-matrix). Student's *t*-tests (p<0.05) or single factor (age) ANOVAs (analysis of variance; p<0.05) combined with post-hoc multiple comparison tests (Tukey-Kramer procedure) were used to compare normalised overlap values across assessed age points. Two factor ANOVAs (subregion identity and age as factors) were used to compare WFA patch or PNN expression across neostriatal subregions and developmental time points.
